# Associations of tissue damage induced inflammatory plasticity in masseter muscle with the resolution of chronic myalgia

**DOI:** 10.1038/s41598-023-49280-1

**Published:** 2023-12-12

**Authors:** Karen A. Lindquist, Sergey A. Shein, Anahit H. Hovhannisyan, Jennifer Mecklenburg, Yi Zou, Zhao Lai, Alexei V. Tumanov, Armen N. Akopian

**Affiliations:** 1 Integrated Biomedical Sciences (IBMS) Program, The School of Medicine, The University of Texas Health Science Center at San Antonio (UTHSCSA), 7703 Floyd Curl Drive, San Antonio, TX 78229-3900 USA; 2 Departments of Endodontics, The School of Dentistry, The University of Texas Health Science Center at San Antonio (UTHSCSA), 7703 Floyd Curl Drive, San Antonio, TX 78229-3900 USA; 3 Departments of Molecular Medicine, The School of Medicine, UTHSCSA, San Antonio, TX USA; 4 Departments of Microbiology, Immunology & Molecular Genetics, The School of Medicine, UTHSCSA, San Antonio, TX 78229 USA; 5 Greehey Children’s Cancer Research Institute, UTHSCSA, San Antonio, TX 78229 USA

**Keywords:** Neuroscience, Physiology, Diseases

## Abstract

Gene plasticity during myogenous temporomandibular disorder (TMDM) development is largely unknown. TMDM could be modeled by intramuscular inflammation or tissue damage. To model inflammation induced TMDM we injected complete Freund’s adjuvant (CFA) into masseter muscle (MM). To model tissue damage induced TMDM we injected extracellular matrix degrading collagenase type 2 (Col). CFA and Col produced distinct myalgia development trajectories. We performed bulk RNA-seq of MM to generate gene plasticity time course. CFA initiated TMDM (1d post-injection) was mainly linked to chemo-tacticity of monocytes and neutrophils. At CFA-induced hypersensitivity post-resolution (5d post-injection), tissue repair processes were pronounced, while inflammation was absent. Col (0.2U) produced acute hypersensitivity linked to tissue repair without inflammatory processes. Col (10U) generated prolonged hypersensitivity with inflammatory processes dominating initiation phase (1d). Pre-resolution phase (6d) was accompanied with acceleration of expressions for tissue repair and pro-inflammatory genes. Flow cytometry showed that immune processes in MM was associated with accumulations of macrophages, natural killer, dendritic and T-cells, further confirming our RNA-seq findings. Altogether, CFA and Col treatments induced different immune processes in MM. Importantly, TMDM resolution was preceded with muscle cell and extracellular matrix repairs, an elevation in immune system gene expressions and distinct immune cell accumulations in MM.

## Introduction

Myogenous temporomandibular disorder (TMDM)^1^ is one of most prevalent types of myofascial pain syndromes^2–4^. Chronic TMDM affects approximately 10% of the population, with two-thirds being women^2^. Precise etiology, pathogenesis, and pathophysiology underlying chronic TMDM remains unclear. Nevertheless, studies showed that TMDM patients could have detectable muscle damage, which is accompanied either no or low-grade inflammation^1,5^. Thus, individual inflammatory mediators, such as tumor necrosis factor-alpha (TNF-α), interleukin-1beta, 6 and 8 (IL-1β, IL-6 and IL-8), in the masseter muscle (MM) were revealed in TMDM patients using a variety of approaches including micro-dialysis^1,5–8^. However, the overall biological processes in MM linked to this tissue damage-generated mild inflammation is largely unknown.

TMDM is thought to develop due to muscle ischemia following consistent and repetitive physical overloading of masticatory muscles^1,5^. Muscle overload can disrupt local blood flow to the muscles of mastication, triggering ischemia, which in turn produces muscle damage, and mild inflammation^9,10^. One way to model TMDM is to mimic intramuscular inflammation generated by standard approaches such as injecting complete Freund’s adjuvant (CFA) into the MM^11,12^. CFA causes severe inflammation, which is similar to myositis leading to such signs as edema, erythema or rubor. The prevailing evidence show that TMDM is seldomly accompanied by myositis^13,14^. Hence, CFA intra-muscular injection does not optimally represent TMDM. Usually, TMDM is accompanied by low inflammation caused by tissue damage during TMDM^14–16^. Muscle damage can be stimulated be tendon ligation, muscle crush, etc. However, such muscle damage is commonly encountered after impact trauma, but not TMDM. Since ischemia-induced muscle damage may involve extracellular matrix (ECM) dysfunction/damage^17–19^ and collagen degradation^20,21^; we generated TMDM-related intramuscular tissue damage by disrupting extracellular matrix with the collagenase type 2 (Col). Col is the main collagenase produced during wound healing and is released during inflammation to direct chemotaxis and degrade tissue components like collagen, proteoglycan, ECM, and cartilage^22–24^. Moreover, elevated levels of Col are seen in TMD patients, especially while experiencing painful episodes^25,26^. We used several dosages of Col and found that 10% of effective dose (ED) corresponded to approximately 0.2U (low dose), while ED_max_ was about 10U (high dose).

Pain conditions in humans and hypersensitivity in animal models develop in phases: initiation or transition to chronic state, maintenance, pre-resolution, resolution and post-resolution^27^. Changes in biological processes during these phases for a variety of hypersensitivity models in mice or pain conditions in humans are largely unknown. This study aimed to bridge a gap in knowledge related to the biological processes in MM during different hypersensitivity phases induced by injections of CFA, low dose (0.2U) Col, or high dose (10U) Col. The biological processes in MM were correlated to distinct hypersensitivity phases via the collection of MM tissue after von Frey mechanical threshold measurements in mice at different time points. We then used bulk RNA seq to trace biological processes within MM. Additionally, certain data obtained from bulk RNA-seq were validated as well as supplemented using flow cytometry and immunohistochemistry, which characterize the immune cell profiles in MM.

## Results:

### CFA-induced transcriptomic/gene changes in the MM

**Figure 1:**
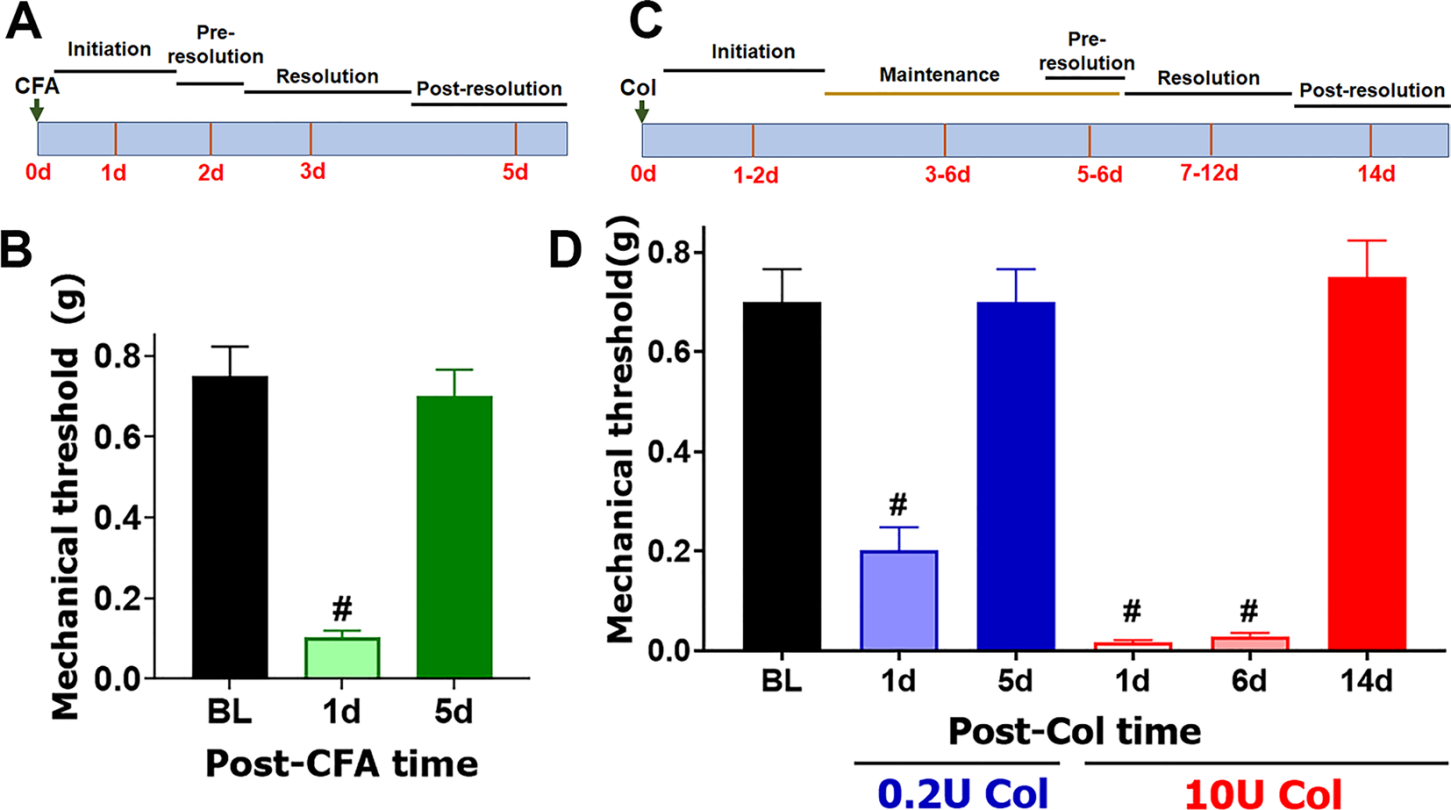
Col- and CFA-induced mechanical hypersensitivity in male mice. (**A**) Schematic representation for pain conditions phases after CFA injection into MM. (**B**) Mechanical hypersensitivity at 1d and 5d post-CFA injected intro-MM in male mice. Statistical analysis is 1-way ANOVA Bonferroni’s post-hoc test (# *P* < 0.0001; n = 8). (**C**) Schematic representation for pain conditions phases after Col injection into MM. (**D**) Mechanical hypersensitivity at 1d and 5d post-0.2U Col and 1d, 6d and 14d post 10U Col injected intro-MM in male mice. Statistical analysis is 1-way ANOVA Bonferroni’s post-hoc test (# *P* < 0.0001; n = 8). X-axis indicates time post injection and injected reagent. Y-axis shows mechanical threshold assessed by von Frey filaments in an orofacial area above MM using up-down approach.

**Figure 2:**
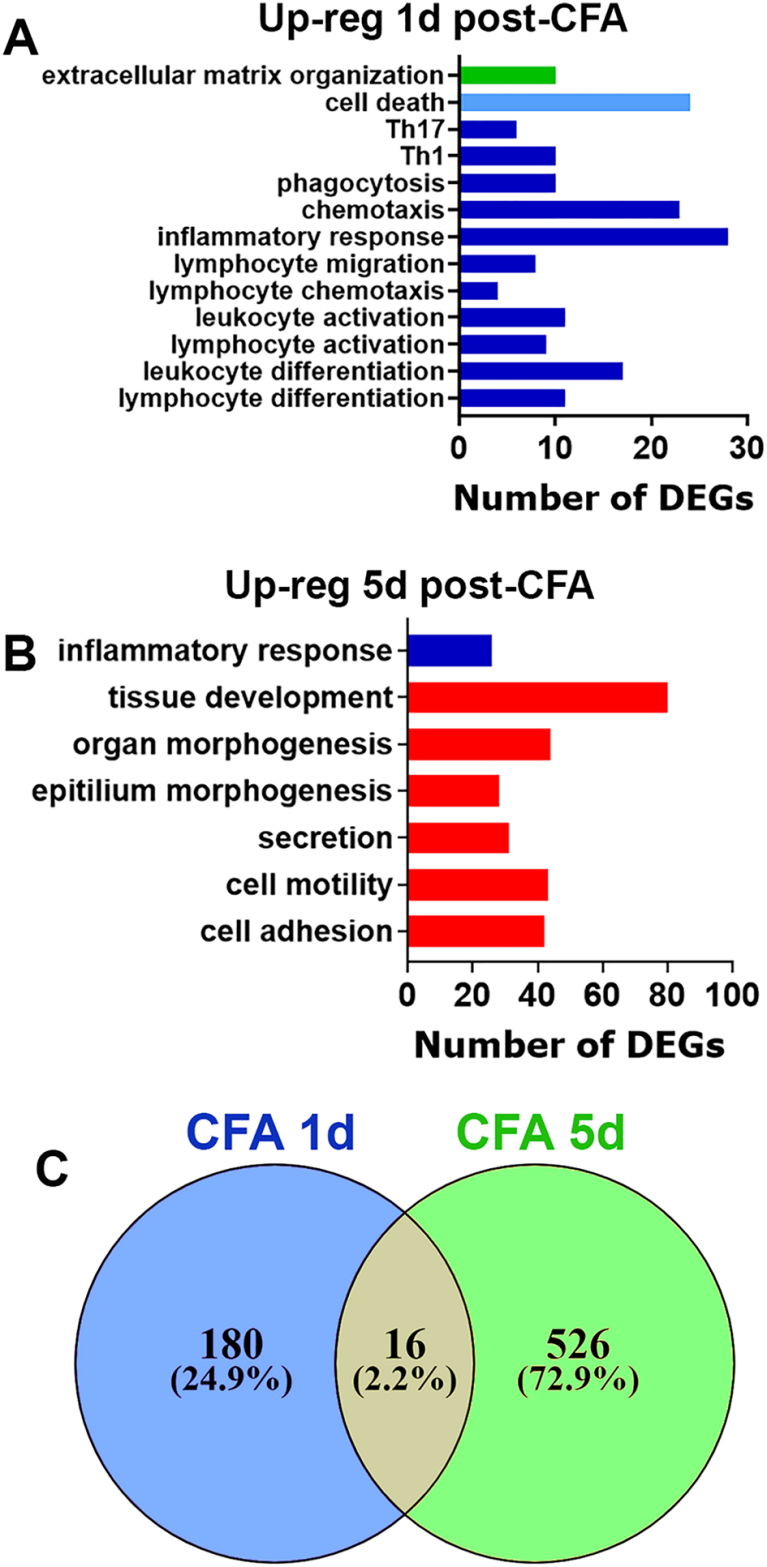
CFA-induced regulation of differential expressed genes (DEGs) in the masseter muscle (MM). (**A**) Biological processes for up-regulated DEGs at 1d post-CFA. (**B**) Biological processes for up-regulated DEGs at 5d post-CFA. (**C**) Venn diagrams of CFA-induced up-regulation of DEGs in the MM at 1d and 5d post-CFA. X-axis on the panels A, B represents numbers of DEGs. Y-axis notes biological processes.

CFA injections in MM produced a distinct trajectory for TMDM development (Fig. 1A). At 1d post-CFA, mice displayed pronounced mechanical hypersensitivity of the orofacial area compared to baseline, which was assessed as previously described (1-way ANOVA; F (2, 21) = 39.12; *P* < 0.0001; n = 8; Fig. 1B)^28^. Immediately after initiation of CFA-induced TMDM, hypersensitivity started to decline to baseline levels (Fig. 1A). At 5d post-CFA, mechanical response thresholds returned to baseline levels (Fig. 1B). We then isolated MM at 1d post CFA (n = 3) and performed bulk RNA-seq as previously described^29^. Considering selections as RPKM > 1, FC > 2 and Padj < 0.05, 196 differentially expressing genes (DEGs) were up-regulated and 78 down-regulated at 1d post CFA treatment. As expected, CFA triggered extensive pro-inflammatory processes in MM at day 1 post injected (Fig. 2A). In contrast, CFA treatment down-regulated DEGs did not fit into biological process according to statistical over-representation test. Among up-regulated DEGs, there are many cytokines, chemokines and their receptors, receptors, such as *Il6, Tnf, Ccl2, Ccr2, Il1b* known to be involved in sensitizing sensory neurons and/or the nociceptive pathway (Table 1). Other notable up-regulated DEGs related to the immune system were *Tlr13, Tlr4, Tlr1, Il33, Csf3, Csf2rb, Fcgr1, Nfkbid, Cd300lf, Selp, Oas2, Irf5, Il2rg*, and *Runx1*. Overall, up-regulated DEGs point toward acute inflammatory processes (*Saa1, Saa3, Tlr4, Tlr1*), chemotactic activity for monocytes (Mo; *Ccl2, Ccr2, Ccl7*), inflammatory monocytes (iMo; *Ccl3, Ccl4, Ccl8, Ccl12*), neutrophils (Neu; *Cxcl2, Cxcl3, Cxcl5, Ccl9*), and low levels of phagocytic activity (*Clec4e, Msr1*). Data also revealed that there were only few DEGs related to macrophage (Mph) and natural killer (NK) cell chemotactic pathways.

**Table 1:** Examples of up-regulated DEGs in MM at 1d post-CFA injection.

ID	BL	CFA 1d	FC	Name
*Cxcl3*	0	80.00	Inf	Chemokine (C–X–C motif) ligand 3
*Cxcl2*	0.3	382.5	1062.4	Chemokine (C–X–C motif) ligand 2
*Saa3*	1.9	1438.1	733.9	Serum amyloid A 3
*Ccl3*	0.9	102.2	107.3	Chemokine (C–C motif) ligand 3
*Saa1*	4.0	315.2	77.1	Serum amyloid A 1
*Ccl7*	8.4	604.9	71.3	Chemokine (C–C motif) ligand 7
*Cxcl5*	2.1	148.3	69.4	Chemokine (C–X–C motif) ligand 5
*Ccl4*	2.2	102.3	44.8	Chemokine (C–C motif) ligand 4
*Il6*	1.2	55.3	44.0	Interleukin 6
*Ccl12*	3.1	99.5	31.8	Chemokine (C–C motif) ligand 12
*Il1b*	19.0	557.5	29.3	Interleukin 1 beta
*Ccl2*	18.4	398.2	21.6	Chemokine (C–C motif) ligand 2
*Clec4n*	6.2	134.4	21.3	C-type lectin domain family 4, member n
*Clec4d*	14.3	305.3	21.2	C-type lectin domain family 4, member d
*Arg1*	39.5	658.9	16.6	Arginase
*Osm*	4.7	74.9	15.7	Oncostatin M
*Msr1*	29.0	296.7	10.2	Macrophage scavenger receptor 1
*Tnf*	5.3	51.1	9.6	Tumor necrosis factor
*Ccl8*	33.6	295.0	8.8	Chemokine (C–C motif) ligand 8
*Ccr5*	32.5	262.7	8.1	Chemokine (C–C motif) receptor 5
*Ccr1*	47.3	301.3	6.4	Chemokine (C–C motif) receptor 1
*Aif1*	21.1	134.0	6.3	Allograft inflammatory factor 1
*Ccl9*	329.0	1840.0	5.6	Chemokine (C–C motif) ligand 9
*Ccr2*	182.0	712.9	3.9	Chemokine (C–C motif) receptor 2

MM was isolated at a post-resolution time point, 5d post CFA (n = 3), and bulk RNA-seq was performed^29^. Using the same selection criteria as above, 542 DEGs were up-regulated and only 18 down-regulated, which did not fit into any biological process (Fig. 2C). Inflammatory processes were substantially resolved. However, a set of DEGs (*Saa3, Ccl3, Aif1*) up-regulated at 1d post-CFA were still present. Additionally, distinct immune system related DEGs compared to 1d post-CFA (*Cxcl17, Cx3cr1, Tlr3, Gzma, Itgax*) were upregulated at 5d post-CFA (Fig. 2B). Generally, as low as 16 upregulated DEGs overlapped when 1d compared to 5d post-CFA time points (Fig. 2C). Biological processes at 5d post-CFA, when mechanical responses were on baseline levels, were dominated by genes involved in various cellular and extracellular matrix repair and developmental processes (Fig. 2B). In summary, biological processes at 1d (mechanical hypersensitivity) and 5d post-CFA (return to baseline nociception level) were found to be significantly different. Transcriptomic changes in the MM triggered at 1d post-injection of CFA were mainly represented by genes controlling chemotactic activity for monocytes and neutrophils. DEGs were linked to tissue repair processes and only few pro-inflammatory genes were up regulated in MM at 5d post administration.

### Low dose (0.2U) collagenase-induced transcriptomic/gene changes in the MM

We used different dosages of Col to produce TMDM hypersensitivity after intra-MM injection. An increase in Col dosage prolonged hypersensitivity (i.e. effect is measured in post-injection days). A 10% effective dose (ED_10_) was generated with approximately 0.2U, while 8, 10 and 15U Col produced similar maximum effect (i.e. longest-lasting hypersensitivity). Hence, EDmax could be any Col dosage between 8 and 15U; we selected 10U. So, tissue damage was mimicked with a low (0.2U) or high (10U) dose MM injections of Col. Low and high Col doses produced dramatically different trajectories for TMDM hypersensitivity development. A low Col dose-induced hypersensitivity development trajectory was similar to CFA (Fig. 1A,D). Low (0.2U) dose Col injections induced mechanical hypersensitivity in orofacial area, which was detected at 1d, but not 6d post injection (1-way ANOVA; F (2, 21) = 23.05; *P* < 0.0001; n = 8; Fig. 1D). High Col dosages generated a long lasting hypersensitivity with distinct phases (Fig. 1C). Thus, at a high dose of Col (10U) we found that mechanical hypersensitivity lasted at least 2 weeks with day 5–6 post-injection as a bifurcation point in the hypersensitivity developmental trajectory (1-way ANOVA; F (3, 28) = 68.00; n = 8; *P* < 0.0001; Fig. 1D).

**Figure 3:**
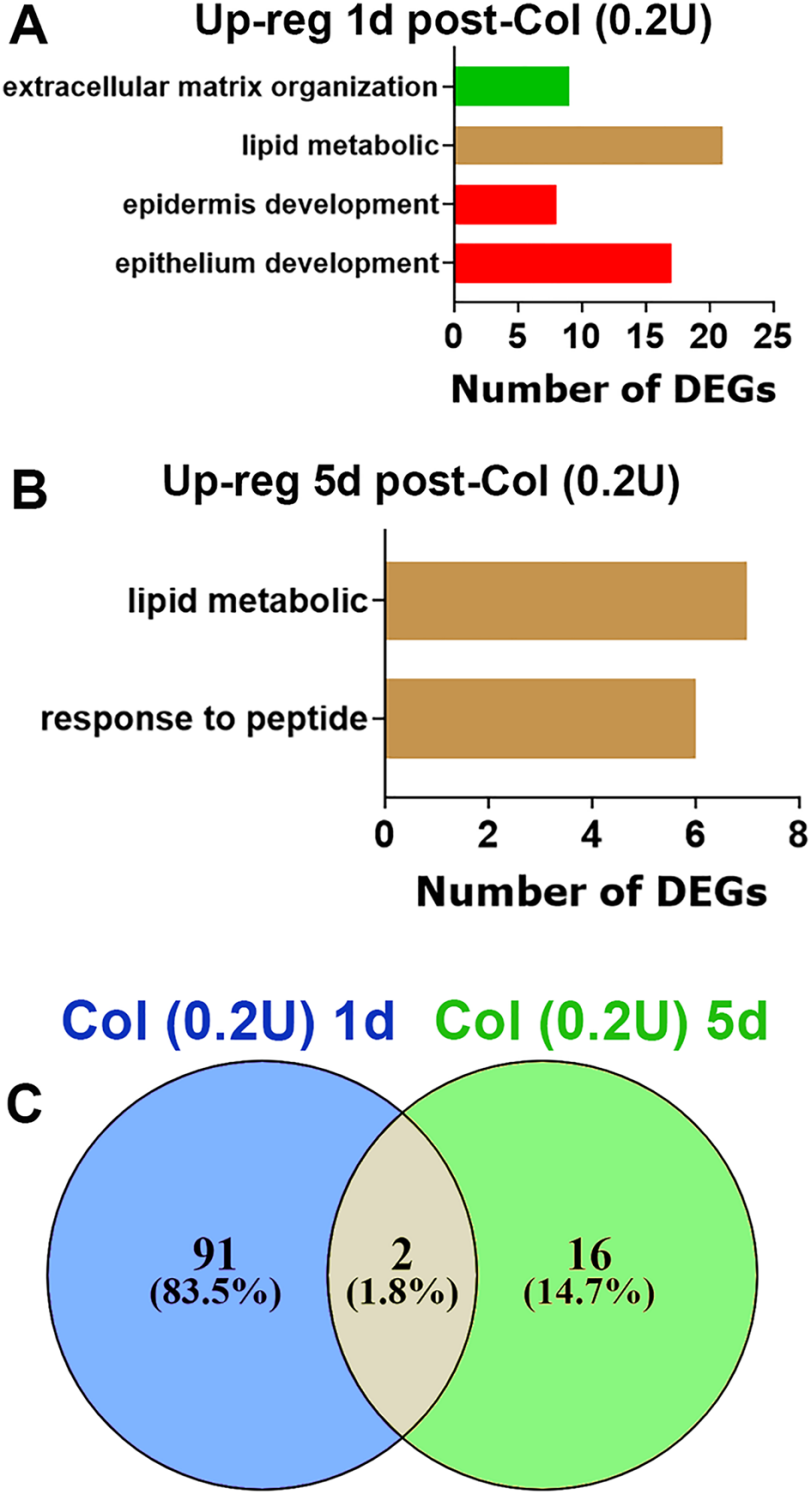
Collagenase type 2 (Col; 0.2U)-induced regulation of DEGs in the MM. (**A**) Biological processes for up-regulated DEGs at 1d post-Col (0.2U). (**B**) Biological processes for up-regulated DEGs at 5d post-Col (0.2U). (**C**) Venn diagrams of Col (0.2U)-induced up-regulation of DEGs in the MM at 1d and 5d post-Col (0.2U). X-axis on the panels A, B represents numbers of DEGs. Y-axis notes biological processes.

We then isolated MM at 1d and 5d post Col (n = 3–4) and bulk RNA-seq was performed as previously described^29^. For 1d post-Col and RPKM > 1, FC > 2 and Padj < 0.05 selection, 93 DEGs were up-regulated and 11 down-regulated (Fig. 3C). Up-regulated DEGs upon 0.2U Col treatment showed biological processes associated with tissue repair, including extracellular matrix reorganization and epithelial cell development, and lipid metabolism (Fig. 3A). Many of the upregulated DEGs related to tissue repair were associated with epidermis development (*Krt14, Ktr15, Krt17, Krt77, Ktr79, Fgfr3, Evpl*) or extracellular matrix organization (*Col9a1, Col9a2, Col9a3, Col10a1, Wnt3a, Mmp20*). Lipid metabolic processes were mainly for membrane lipid biosynthesis processes of sphingolipid biosynthetic process (*Abca12*, *Pnpla1, Elovl3, Elovl4, Elovl6*) and fatty acid biosynthetic process (*Alox12E, Elovl3, Elovl4, Elovl6*) (Fig. 3A). Sphingolipids and fatty acids play a vital role in orchestrating the inflammatory response and consequently the development of pain/hypersensitivity in many conditions (more than 4000 citations, including^30–32^). Interestingly, low Col dosage did not trigger upregulation of inflammatory processes at 1d post-administration (Fig. 3A). Down regulated DEGs by 0.2U Col did not highlight any biological process according to a statistical over-representation test. At 5d post-Col, only 18 DEGs were up-regulated and 8 down-regulated. Among these up-regulated 18 DEGs, 5 were related to fatty acid metabolic process (*Adipod, Fasn, Scd1, Pck1 and Elovl6;* Fig. 3B). It seemed that the repair process was completed by 5d post-Col (0.2U) (Fig. 3B). Only two DEGs remained up-regulated (*Ucp1, Elovl6*) and only two down-regulated (*Slc2a3, Hs3st5*) after 5d post-Col (*Supplementary material*). Overall, low dosage of Col (0.2U) produced acute orofacial hypersensitivity which was linked to tissue repair, but not inflammatory processes. These results suggest that tissue restoration was fully completed 5 days post injection.

### High dose (10U) collagenase-induced transcriptomic/gene changes in the MM

**Figure 4:**
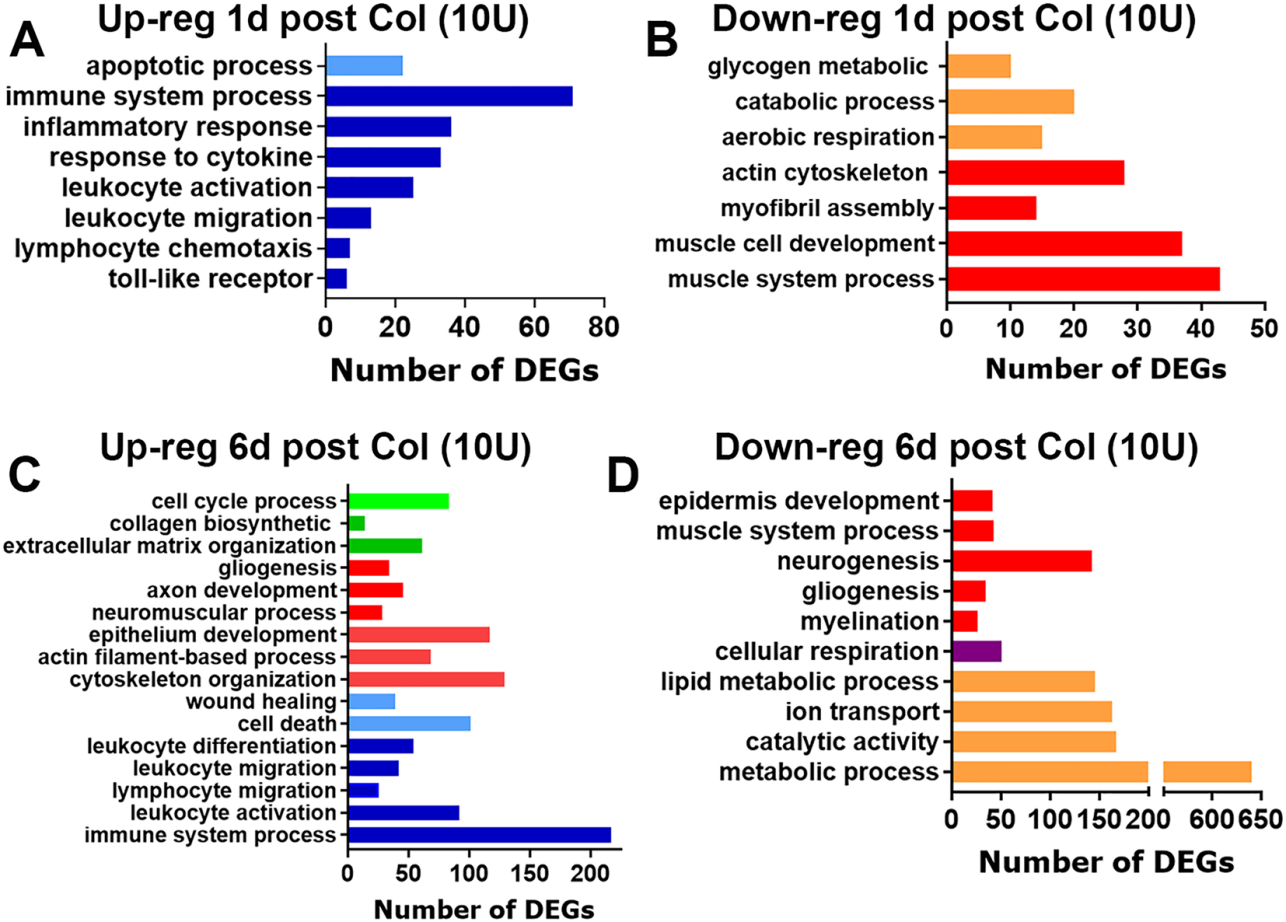
Up- and down-regulated biological processes in the MM after 10U Col treatments. (**A**) Biological processes for up-regulated DEGs at 1d post-Col (10U). (**B**) Biological processes for down-regulated DEGs at 1d post-Col (10U). (**C**) Biological processes for up-regulated DEGs at 6d post-Col (10U). (**D**) Biological processes for down-regulated DEGs at 6d post-Col (10U). The X-axis on the panels A-D represents numbers of DEGs. The Y-axis notes biological processes.

According to observed development of high dose-induced TMDM hypersensitivity, we isolated MM (n = 3–4) at 1d, 6d and 14d post Col (10U) and evaluated transcriptomic at these time points. DEGs selection criteria was as above. At 1d post-Col, 334 DEGs were up and 464 DEGs were down-regulated. Inflammatory processes dominated up-regulated DEGs at 1d post-Col (Fig. 4A). Down-regulated DEGs were associated with muscle damage and reductions of aerobic cellular respiration (Fig. 4B). At 6d post-Col, as many as 1623 DEGs were up, and 1648 DEGs down-regulated. Analysis showed that at 6d post-Col, which is the last time point when hypersensitivity was detected, inflammation was elevated compared to 1d post-injection, tissue repair had begun and lipid metabolism was reduced, while cellular respiration and metabolism, especially lipid metabolism, had yet to be recovered (Fig. 4C,D). Upon orofacial myogenous mechanical hypersensitivity resolution at 14d post-Col, only 99 DEGs were up- and 65 down-regulated. It appeared that biological processes returned to homeostasis and none of them was up or down-regulated.

**Figure 5:**
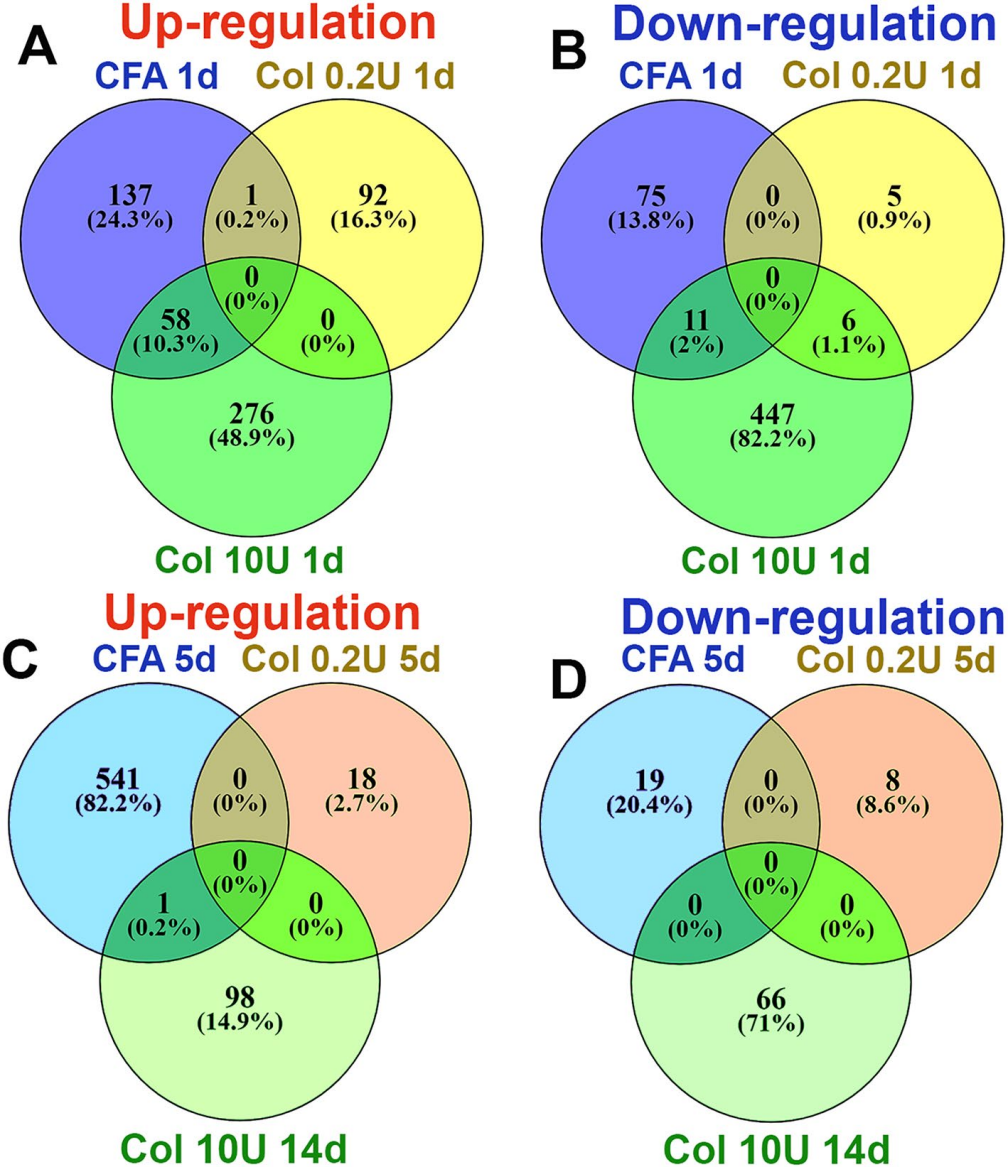
Comparisons of up- and down-regulated DEGs in the MM after CFA, 0.2U Col or 10U Col treatments. (**A**) Venn diagrams of CFA, 0.2U Col and 10U Col-induced up-regulation of DEGs in the MM at 1d post-treatments. (**B**) Venn diagrams of CFA, 0.2U Col and 10U Col-induced down-regulation of DEGs in the MM at 1d post-treatments as indicated on panels. (**C**) Venn diagrams of CFA, 0.2U Col and 10U Col-induced up-regulation of DEGs in the MM at 5d post-treatments. (**D**) Venn diagrams of CFA, 0.2U Col and 10U Col-induced down-regulation of DEGs in the MM at 5d post-treatments as indicated on panels.

Next, we analyzed differences in inflammatory mediators (cytokines and chemokines) and processes at 1d post-injection of Col compared to CFA. CFA, 0.2U Col, and 10U Col initially (1d post-injection) produced substantially different expression changes in MM (Fig. 5A,B). However, like CFA, 10U Col treatment up-regulated certain numbers of the same pro-inflammatory genes (Table 2). Thus, common up-regulated DEGs for CFA and 10U Col intramuscular treatments included monocyte and neutrophil associated genes: *Cxcl2, Cxcl3, Cxcl5, Ccl2, Ccl7, Ccl8, Ccl12, Ccr5, Ccr2, Aif1, Mrc1, Msr1, Ms4a6b, Clec4d, Clec4n, Fcgr1, Mt2, Mcoln2, Runx1, Tlr1, Tlr4*, etc. One of the main differences in biological processes at 1d post-injection between CFA and 10U Col treatment was that, unlike CFA, 10U Col administration led to up-regulation of genes linked to macrophage activation and the down-regulation of DEGs associated with muscle structure, muscle cell development and catabolic processes (Fig. 4B).

**Table 2:** Examples of up-regulated DEGs in MM at 1d post-CFA or Col (10U) injection.

CFA	Col	Col & CFA	Name
*Cxcl3*	*Cxcl3*	+	Chemokine (C–X–C motif) ligand 3
*Cxcl2*	*Cxcl2*	+	Chemokine (C–X–C motif) ligand 2
*Saa3*	*Saa3*	+	Serum amyloid A 3
*Ccl3*	*Ccl3*	+	Chemokine (C–C motif) ligand 3
*Saa1*	–	−	Serum amyloid A 1
*Ccl7*	*Ccl7*	+	Chemokine (C–C motif) ligand 7
*Cxcl5*	*Cxcl5*	+	Chemokine (C-X-C motif) ligand 5
*Ccl4*	–	−	Chemokine (C–C motif) ligand 4
*Il6*	–	−	Interleukin 6
*Ccl12*	*Ccl12*	+	Chemokine (C–C motif) ligand 12
*Il1b*	–	−	Interleukin 1 beta
*Ccl2*	*Ccl2*	+	chemokine (C–C motif) ligand 2
*Clec4e*	–	−	C-type lectin domain family 4, member e
*Clec4n*	*Clec4n*	+	C-type lectin domain family 4, member n
*Clec4d*	*Clec4d*	+	C-type lectin domain family 4, member d
*Arg1*	–	−	Arginase
*Osm*	–	−	Oncostatin M
*Msr1*	*Msr1*	+	Macrophage scavenger receptor 1
*Tnf*	–	−	Tumor necrosis factor
*Ccl8*	*Ccl8*	+	Chemokine (C–C motif) ligand 8
*Ccr5*	*Ccr5*	+	Chemokine (C–C motif) receptor 5
*Ccr1*	–	−	Chemokine (C–C motif) receptor 1
*Aif1*	*Aif1*	+	Allograft inflammatory factor 1
*Ccl9*	–	−	Chemokine (C–C motif) ligand 9
*Ccr2*	*Ccr2*	+	Chemokine (C–C motif) receptor 2
*Msr1*	*Msr1*	+	Macrophage scavenger receptor 1
*Ms4a6b*	*Ms4a6b*	+	Membrane-spanning 4-domains, subfam A, m-6B
*Fcgr1*	*Fcgr1*	+	Fc receptor, IgG, high affinity I
*Runx1*	*Runx1*	+	Runt related transcription factor 1
*Tlr1*	*Tlr1*	+	Toll-like receptor 1
*Tlr4*	*Tlr4*	+	Toll-like receptor 4
–	*Tlr7*	−	Toll-like receptor 7
*Tlr13*	–	−	Toll-like receptor 13
–	*CD68*	−	CD68 antigen
–	*CD163*	−	CD163 antigen

**Figure 6:**
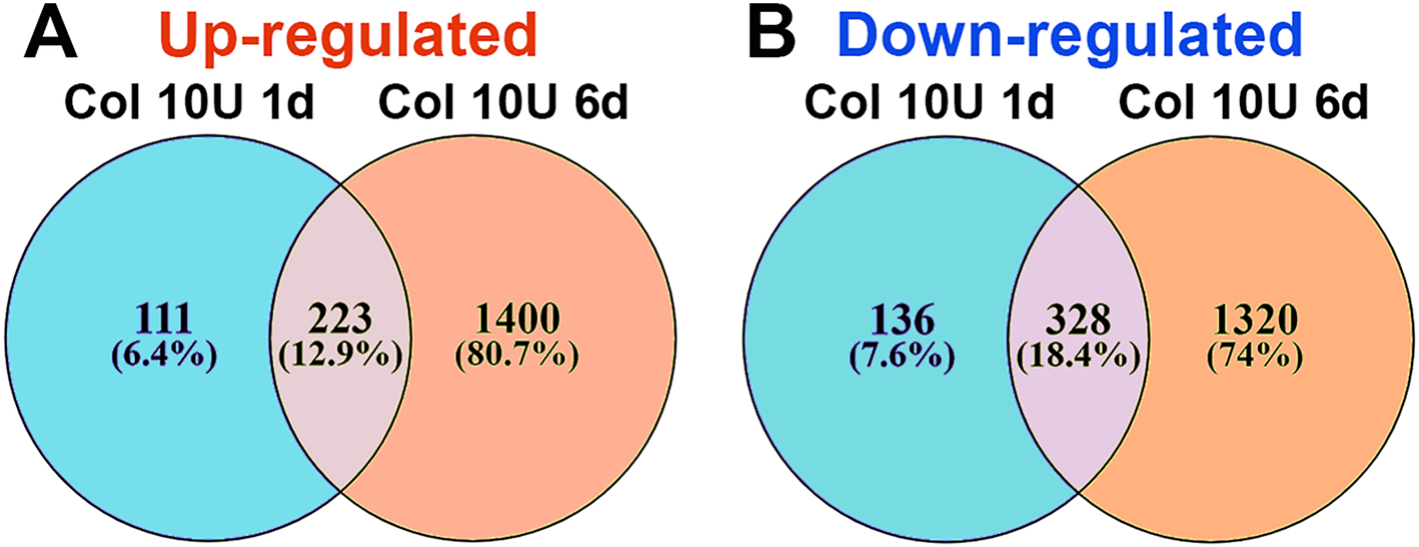
Comparisons of up- and down-regulated DEGs in the MM at different time points after 10U Col treatments. (**A**) Venn diagrams of 10U Col-induced up-regulation of DEGs in the MM at 1d and 6d post-treatments. (**B**) Venn diagrams of 10U Col-induced down-regulation of DEGs in the MM at 1d and 6d post-treatments as indicated on panels.

At the time point preceding myalgia resolution (5-6d for 10U Col; Fig. 1C), expression and the number of pro-inflammatory DEGs were significantly increased compared to 1d post 10U Col (Fig. 4C,D). The rise in pro-inflammatory DEG numbers accompanied wound healing and tissue repair processes, included partial muscle, glia, neuro-muscular junction, extracellular matrix, and epithelium repair (Fig. 4C), as well as drastic downregulation of metabolic processes (Fig. 4D). Overall, expression changes in MM from 1 to 6d post-Col were dramatic (Fig. 6A,B). Next, we looked for a subset of immune system related DEGs that could be upregulated at 6d compared to 1d post-Col (10U). Thus, at 6d post-Col, biological processes in MM included accumulation of macrophages (*Cx3cr1, Ccl22, Mpeg1, lyz2, Csfr1*), natural killer (NK; *granzymes, CD53, Cd244*) and natural killer T cells (NKT; *Cxcl16*), dendritic cells (*Itgax, Cd48, Cd80, Cd86*) and T-cells (*Cd4, Cd40, Cd72*) (Table 3).

**Table 3:** Examples of up-regulated DEGs in MM at 6d compared to 1d post-Col (10U).

Col 1d	Col 6d	Col 1d & Col 6d	Name
*–*	*Gzme*	−	Granzyme E
*–*	*Gzmb*	−	Granzyme B
*Cxcl3*	*–*	−	Chemokine (C–X–C motif) ligand 3
*Ccl3*	*Ccl3*	+	Chemokine (C–C motif) ligand 3
*–*	*Cx3cr1*	−	Chemokine (C–X3–C motif) receptor 1
*Ccl7*	*Ccl7*	+	Chemokine (C–C motif) ligand 7
*–*	*Cxcl16*	−	Chemokine (C–X–C motif) ligand 16
*Ccl12*	*Ccl12*	+	Chemokine (C–C motif) ligand 12
*Ccl2*	*Ccl2*	+	Chemokine (C–C motif) ligand 2
*–*	*IL6*	−	Interleukin 6
*–*	*Il1b*	−	Interleukin 1 beta
*–*	*Il21r*	−	Interleukin 21 receptor
*Clec4n*	*Clec4n*	+	C-type lectin domain family 4, member n
*Clec4d*	*Clec4d*	+	C-type lectin domain family 4, member d
*–*	*Ccl5*	−	Chemokine (C–C motif) ligand 5
*Mrc1*	*Mrc1*	+	Mannose receptor, C type 1
*Msr1*	*Msr1*	+	Macrophage scavenger receptor 1
*–*	*Mpeg1*	−	Macrophage expressed gene 1
*Ccl8*	*Ccl8*	+	Chemokine (C–C motif) ligand 8
*Ccr5*	*Ccr5*	+	Chemokine (C–C motif) receptor 5
*–*	*Ccl22*	−	Chemokine (C–C motif) ligand 22
*–*	*Ccr7*	−	Chemokine (C–C motif) receptor 7
*Aif1*	*Aif1*	+	Allograft inflammatory factor 1
*–*	*Lyz2*	−	Lysozyme 2
*–*	*Csf1r*	−	Colony stimulating factor 1 receptor
*Ccr2*	*Ccr2*	+	Chemokine (C–C motif) receptor 2
*–*	*Tnfsf8*	−	TNF superfamily, member 8
*–*	*Tnfrsf23*	−	TNF receptor superfamily, member 22
*Ms4a6b*	*Ms4a6b*	+	Membrane-spanning 4-domains, subfam A, m-6B
*Tlr1*	*Tlr1*	+	Toll-like receptor 1
*-*	*Tlr2*	−	Toll-like receptor 2
*Tlr4*	*Tlr4*	+	Toll-like receptor 4
*Tlr7*	*Tlr7*	+	Toll-like receptor 7
*–*	*Tlr8*	−	Toll-like receptor 8
*–*	*Tlr9*	−	Toll-like receptor 9
*–*	*Itgax (Cd11c)*	−	Integrin alpha X
*–*	*Cd4*	−	CD4 antigen
*–*	*Cd14*	−	CD14 antigen
*–*	*Cd44*	−	CD44 antigen
*–*	*Cd48*	−	CD48 antigen
*–*	*Cd53*	−	CD53 antigen
*CD68*	*Cd68*	+	CD68 antigen
*–*	*Cd72*	−	CD72 antigen
*–*	*Cd74*	−	CD72 antigen
*–*	*Cd80*	−	CD80 antigen
*Cd84*	*Cd84*	+	CD84 antigen
*–*	*Cd86*	−	CD86 antigen
*CD163*	*–*	−	CD163 antigen
*–*	*Cd248*	−	CD248 antigen
*–*	*C3ar1*	*−*	Complement component 3a receptor 1

Besides, direct regulation immune system associated genes, at 6d post-Col (10U), many lipid metabolism genes were down regulated (Fig. 4D). Down-regulated groups of DEGs include acetyl-CoA metabolic process (*Acacb, Acaa2, Acss2, Hmgcs2, Mlycd, Auh, Ivd, Gcdh*), neutral lipid catabolic process *(Lpl, Lipe, Faah, Dagla*), fatty acid derivative metabolic process (*Dgat2, Abhd16a, Dbp*), and fatty acid oxidation (*Adipod, Bdh2, Lep, Etfb, Dbp*). These processes could add to escalation and tissue repair at this time point in development of TMDM.

We next evaluated gene changes at 14d post-10U Col versus 5d post-0.2U Col. At these time points, hypersensitivity returned to baseline for both 0.2U and 10U treatments (Fig. 1C,D). Few DEGs were up- and down-regulated at these time points. Among those few regulated DEGs for 0.2U and 10U treatments, almost no similarity could have been detected (Fig. 5C,D). In summary, CFA and Col treatment induced substantially different biological processes in the MM. Additionally, the transcriptomic analysis showed that mechanical hypersensitivity resolution correlates with gradual muscle and extracellular matrix repair, which was supplemented by an elevation in immune system gene expression compared to 1d post 10U Col.

### Collagenase-induced alterations in immune cell profiles in masseter muscle (MM)

**Figure 7:**
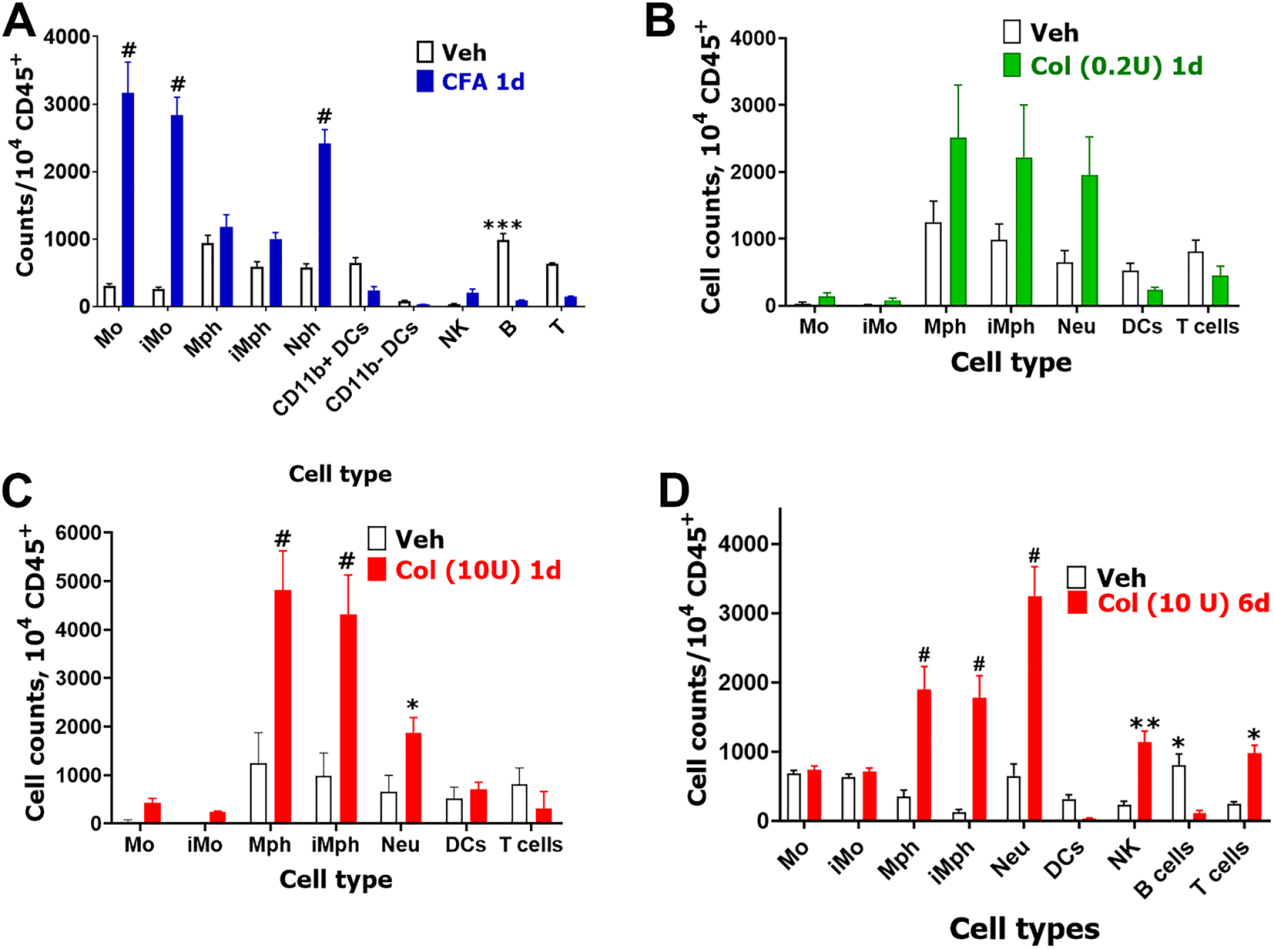
Immune cell profiles in CFA and Col treated MM. (**A**) Immune cell counts per 104 CD45 + cells in the MM at 1d post-vehicle (Veh) or CFA single intramuscular treatment. (**B**) Immune cell counts per 104 CD45 + cells in MM at 1d post-vehicle (Veh) or Col (0.2U) single intramuscular treatment. (**C**) Immune cell counts per 104 CD45 + cells in MM at 1d post-vehicle (Veh) or Col (10U) single intramuscular treatment. (**D**) Immune cell counts per 104 CD45 + cells in MM at 6d post-vehicle (Veh) or Col (10U) single intramuscular treatment. DCs—dendritic cells; Mph—macrophages; iMph—inflammatory macrophages; Mo – monocytes; iMo—inflammatory monocytes; NK—natural killer cells; B–B-cells; T–T-cells and Neu—neutrophils. Statistic is 2-way ANOVA (*P < 0.05; **P < 0.01; ***P < 0.001; #*P* < 0.0001; n = 3–6).

To validate bulk RNA-seq data, we used flow cytometry to evaluate immune cell (CD45^+^) profiles in MM at different time points after CFA or Col injections (Suppl Fig. 1). CFA intra MM treatment increased CD45^+^ cell numbers > 14-fold at 1d post-CFA (*t*-test; Veh vs CFA; 2266 ± 77 vs 29,300 ± 6084, t = 4.443, df = 4; *P* = 0.011; n = 3). This increase in CD45^+^ cells was due to infiltration/proliferation of monocytes (Mo), inflammatory monocytes (iMo), and neutrophils (Neu) in MM, while the number of B-cells were reduced (2-way ANOVA; interaction F (9, 40) = 42.42; *P* < 0.0001; n = 3; Fig. 7A, Suppl Fig. 1). Mph, inflammatory Mph (iMph) as well as natural killer (NK), dendritic cells (DC) and T-cells numbers were not significantly changed at 1d post-CFA (Fig. 7A).

Low dose (0.2U) injection of Col slightly increased numbers of CD45^+^ cells, which was statistically significant (*t*-test; Veh vs 0.2U Col; 4965 ± 1040 vs 9928 ± 3671, t = 1.301, df = 6; *P* = 0.24; n = 4, Fig. 7B). This slight increase was caused by infiltrations/proliferations of macrophages (Mph), inflammatory macrophages (iMph) and Neu in MM, but was statistically insignificant at 1d post-administration (2-way ANOVA; interaction F (6, 42) = 2.24; *P* = 0.058; n = 4; Fig. 7B). An increase in CD45^+^ cells 1d after 10U Col intra-MM injection was substantial, but not as pronounced compared to CFA treatment (*t*-test; Veh vs 10U Col; 2240 ± 777 vs 14,980 ± 2648, t = 4.105, df = 6; *P* = 0.0063; n = 4). Moreover, unlike CFA, 10U Col elevated infiltration/proliferation of Mph, iMph and Neu, while Mo and iMo were unaffected in MM (2-way ANOVA; interaction F (6, 42) = 29.70; *P* < 0.0001; n = 4; Fig. 7C).

Since 6d post Col (10U) treatment is a critical time point for the development of chronicity in orofacial hypersensitivity, we performed flow cytometry on MM tissue at 6d-post Col injection and found substantially increased CD45^+^ cell counts (*t*-test; Veh vs 10U Col; 3620 ± 113 vs 9980 ± 337, t = 18.87, df = 6; P < 0.0001; n = 4). Besides infiltration of Mph, iMph and Neu at 1d post Col, 6d post-10U Col elevated NK and T cells (2-way ANOVA; interaction F (8, 54) = 19.37; *P* < 0.0001; n = 4; Fig. 7D). These data verify bulk RNA-seq results, which implied elevation of Mph, iMph and Neu as well as NK and T cells at 6d post-Col (10U).

Finally, we performed flow cytometry on MM tissue after resolution of orofacial hypersensitivity for CFA-treatment (resolution found to be at 5d post-injection) and Col-treatment (0.2U or 10U, resolution was at 5d and 14d post-administration, respectively) (Figs. 1A–D). After CFA-induced hypersensitivity resolution, no elevation of CD45^+^ was detected in MM compared to vehicle treatment (*t*-test; Veh vs CFA; 4125 ± 728 vs 6944 ± 564, t = 0.3000, df = 7; *P* = 0.77; n = 3–6). Examination of different immune cell types showed that changes in immune cell profiles at 5d post-CFA compared 5d post vehicle were insignificant (Suppl Fig. 2A). Similar to CFA, 14d-post Col 0.2U injection led to no increase in CD45^+^ cell counts (*t*-test; Veh vs 0.2U Col; 558 ± 90 vs 657 ± 229, t = 0.4623, df = 8; *P* = 0.66 n = 4–6). As expected, immune cell profiles did not undergo changes (1-way ANOVA; F (6, 56) = 1.254; F (6, 56) = 1.254; *P* = 0.29; n = 4–6; Suppl Fig. 2B). However, MM at 14d post 10U Col injection still had a statistically significant increase of CD45^+^ cell counts (*t*-test; Veh vs 10U Col; 558 ± 90 vs 1450 ± 196, t = 4.639, df = 8; *P* = 0.0017; n = 4–6). Elevation of CD45^+^ was mainly due to Neu, which remained elevated in MM at 14d post-10U Col injection (2-way ANOVA; interaction F (6, 49) = 8.201; *P* < 0.0001; n = 4–6; Suppl Fig. 2B). In summary, CFA-induced inflammation was principally different compared to Col-triggered inflammation. When compared to a 10U dose of Col, CFA produced a significant elevation of different types of myeloid cells at the initial stage (1d post-injection) (Fig. 7). Furthermore, the pre-resolution stage (6d) after Col (10U) treatment was characterized by significant activation and infiltration/proliferation of myeloid cells as well as NK and T cells in MM (Fig. 7D). Finally, at 14d post-Col, when mechanical sensitivity was resolved (Fig. 1C,D), the number of CD45^+^ cells in MM did not return to vehicle-treated levels due to Neu, which remained elevated (Suppl Fig. 2B).

**Figure 8:**
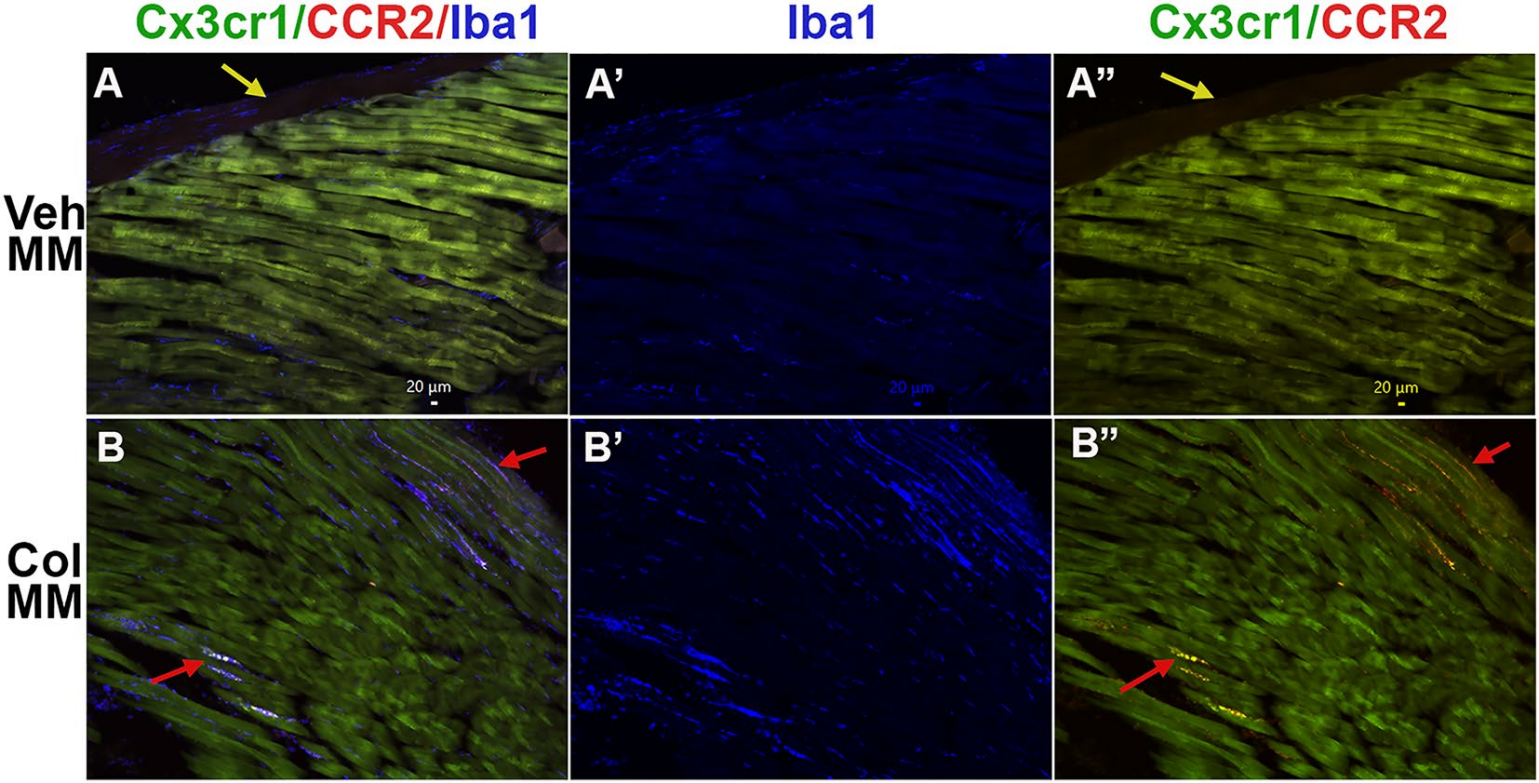
Expression of macrophage/monocyte markers in the MM treated with Col (10U). (**A**, **B”**) The MM of Cx3cr1-GFP/CCR2-RFP reporter mice intramuscularly treated with Veh (Veh MM; panels **A**–**A**”) or 10U Col (Col MM; panels **B**–**B**”) labeled with Iba1. Labeling on each panel is indicated. Yellow arrows on panels A and A” point to the fascia. Red arrows on the *panels B* and *B”* point to immune cells localized between muscle fibers.

To further validate our sequencing and flow cytometry data, immunohistochemistry (IHC) was used. CFA induced accumulation of Mo, while Col increased Mph and Neu. Hence, we defined regulation of immune cell populations in MM with 10U Col, but not CFA. Since our recent showed presence of several residential macrophage (Mph) and neutrophil (Neu) at periphery, we have used following appropriate markers: CX3CR1, CCR2, Iba1 (aka *Aif1*) and S100a8^33^. Accordingly, vehicle or Col (10U) was injected into MM of Cx3cr1-GFP/Ccr2-RFP reporter mice. One day post-injection MM were dissected and used for IHC labeling for Iba1 (marker for Mph) or S100a8 (marker for Neu)^34^. CCR2, CX3CR1, and S100a8 were almost absent in the MM of vehicle-injected mice (Fig. 8A,A”; data not shown). Low numbers of Iba1^+^ cells were detected between muscle fibers in control mice (Fig. 8A,A’). Col injection into the MM triggered the appearance of large numbers of CCR2^+^, CX3CR1^+^, and S100a8^+^ cells (Fig. 8A,A” vs B, B”, data not shown), as well as a visible up-regulation of Iba1^+^ cells (Fig. 8A,A’ vs B,B’). Altogether, these results showed that intra-MM Col injection up-regulated multiple types of myeloid cells in the MM.

## Discussion

The pathogenesis of myogenous temporomandibular disorder (TMDM) remains largely unknown. However, there is widespread opinion that TMDM is a consequence of tissue damage caused by consistent, repetitive, and prolonged physical overload of the muscles of mastication leading to ischemia^1,5,10^. Tissue damage accompanying muscle ischemia leads to mild inflammation that is characterized by release of inflammatory mediators, such as neuropeptides, serotonin (5-HT), and cytokines that may activate and sensitize nociceptors on peripheral sensory afferents to induce muscle pain and allodynia^35^. Microdialysis sampling mediators in MM showed that IL-1β, GM-CSF, IL-6, IL-7, IL-8 and IL-13 were higher in TMDM patients^1^. Other small sample sized studies confirmed these findings on significant elevation of cytokines in active trigger points of muscles during myalgia^8,36^. However, the largest sample sized studies found no elevation in cytokines levels during chronic myalgia ^6,7^. Overall, there is a consensus that chronic and persistent TMDM correlates with none to mild inflammation. However, there is no clear picture on what type of inflammation and biological processes are present in masticatory muscles. In this regard, we mimicked TMDM inflammation two ways: a direct induction with CFA, which leads to pronounced unusual for TMDM inflammation, or recreating tissue damage in MM by disrupting the extracellular matrix with collagenase type-2 (Col). Next, we evaluated transcriptional changes in MM at different time points corresponding to distinct phases of the developing TMDM hypersensitivity. Furthermore, using flow cytometry and/or IHC, we examined infiltration/proliferation of immune cells at the same time points after CFA or Col injections. These several approaches allowed us to draw conclusions on the biological processes occurring in MM after CFA or Col injections, and correlated these processes to specific phases in the development of hypersensitivity.

To focus this study, we concentrated on understanding the biological processes in the MM of males, who represent as many as 1/3 TMDM patients. CFA-induced orofacial hypersensitivity was transient and lasted up to 5d. When hypersensitivity resolved at 5d, biological processes related to tissue repair were still present. However, inflammatory processes were mostly receded. In contrast, at the initial stage, (1d post-CFA) hypersensitivity was apparent, and inflammation was the dominant biological process in MM. At this stage post-CFA, active immune cells were composed of infiltrating monocytes (Mo), inflammatory monocytes (iMo) and neutrophils (Neu). Importantly, bulk RNA-seq and flow cytometry data indicate that CFA triggers minimal (if any) changes in macrophage activity. A similar pattern in the immune system behavior was noted after CFA injection into the paw^15^. Nevertheless, there are multiple publications reporting prolonged hypersensitivity after CFA injection. It is presumed that the effect of CFA is batch/lot dependent. However, it is still not clear why some CFA batches/lots produce chronic pain, while other CFA batches/lots induce only an acute pain condition.

Treatment with low dose Col generated weak/low inflammation with slight up-regulation of *Cd68, Ccl2, Ccl7*, but not pro-inflammatory interleukins, such as IL-1β and IL-6. It is likely that 10U Col is more representative of MM damage than 0.2U Col. Moreover, 0.2U Col did not trigger substantial inflammatory processes, while 10U Col injection produced drastic activation and/or infiltration of immune cells. Unlike CFA, dominant cells were macrophages (Mph) and inflammatory macrophages (iMph) alone with Neu at 1d post-Col (10U). Similar behavior of the immune system was observed in the paw after incision surgery^15^. Another key difference of Col (10U) compared to CFA treatments was a significant Col-triggered down-regulation of genes necessary for muscle tissue repair, including *Casq1, Myom2, Myh7, Mylk2, Myoz1, Lmod1, Acta1, Actn3, Smtn, Tpm1, Tcap, Tmod4, Tnnt3, Tuba8*, etc^37–40^.

TMDM is a chronic pain condition^41^. Hypersensitivity after single treatment with 10U Col was eventually resolved by 12-14d. Five to six days post Col treatment is a critical bifurcation point preceding pain resolution (Figs. 1C,D). Hence, understanding the biological processes in MM at this period/pain condition phase is important. Compared to 1d post-Col, at 6d post-Col, up-regulation of DEGs related to tissue repair, reduction of lipid metabolism and elevation of immune system related gene expressions in MM was noted. Up-regulation of *Cx3cr1, Ccl22, mpeg1, lyz2, csfr1* indicated an additional activation and/or accumulation of pro-inflammatory macrophages^42,43^. *Cxcl16* is produced by dendritic cells and is chemoattractant for NKT cells^44^. *Granzymes, CD53 and Cd244* up-regulation implies activation/infiltration of NK cells^45,46^. *Itgax* (aka *CD11c*), as well as *Cd48, Cd80,* and *Cd86* relate to DC^47,48^. Finally, *Cd4* and *Cd40* are associated with T-cells^49^. Overall, some immune system related genes were present at 1d post-Col, but their expression was dramatically increased at 6d post-Col. These genes are mainly associated with macrophage activation. Moreover, from 1 to 6d post-10U Col, we observed elevation of genes linked to activation/accumulation of NK, NKT and T-cells.

The inflammatory response ignited by activation of immune cells and supported by changes in lipid metabolism could play a central role in bridging the initial response to muscle injury to that of timely muscle injury repare^50^ or lead to persistent tissue damage^51,52^, which contributes to chronic TMDM. Muscle damage induces three pathways that activate inflammatory cells: complement cascade (C3a and C5a) that recruits circulating leukocytes; damage-associated molecular patterns (DAMPs), responsible for recruiting circulating leukocytes to the injured site; and muscle-activated local immune cells releasing cytokines and chemokines^40^. We reported here that these processes took place at 1d and/or 6d post-Col (10U) injection into MM (Table 3). The cytokines CCL2, CCR2 and some TLRs were present at both time points^53^ (Table 3), while CXCL2, IL-1β, C1 and C3 complex components and chemokines, CDs and TLR were encountered only at certain time points ^54^ (Table 3). It has been suggested that muscle damage-attracted macrophages clean tissue via phagocytosis, which in turn convert macrophages into healing macrophages driving myoblast fusion and growth, fibrosis, vascularization, and return to homeostasis^55,56^. TNF-α and IL-1β induce the proliferation and differentiation of myoblasts^57^. IL-1β decreases the level of myostatin, a negative regulator of muscle growth ^58^. Despite being considered proinflammatory mediators, ablation of TNF-α or IL-6 displays poor muscle regeneration^50^. The CCR2-CCL2 pathway is another pro-inflammatory pathway that promotes macrophage recruitment, insulin-like growth factor-1 (IGF-I) production and stimulation of muscle regeneration^59^. Similarly, blockage of pro-inflammatory TLR4 in knock-out mice resulted in severe muscle injury^60^. Importantly, in contrast, mild inflammation with low TNF-α and scarce macrophage infiltration can lead to poor muscle regeneration^61^. Our results are consistent with these multiple findings pointing to an important role of escalating inflammation in muscle recovery from injury and resolution of TMDM, while mild inflammation creates a negative environment for muscle regeneration. It was also suggested that M2 macrophages may stimulate myogenic precursor cell commitment into differentiated myocytes and the formation of mature myotubes^62^. However, our data indicated that IL-10 and IL-4 were not upregulated in MM by CFA, 0.2U and 10U Col at any time point.

In conclusion, based on our findings, we favor the hypothesis that escalating inflammation in MM is critical to resolve TMDM hypersensitivity triggered by tissue damage mimicking Col intra-MM injection. Our results advance our understanding of the mechanisms controlling TMDM chronicity and provide an informatic basis for further studies on the regulation of gene plasticity in muscle affected by myalgia. The molecular basis of mechanisms controlling chronicity is only starting to emerge from preclinical and clinical studies but there is already evidence that chronic pain resolution involves pro-inflammatory immune cell types, which traditionally considered pro-pain^40,63–66^.

## Materials and methods

### Ethical approval

The reporting in the manuscript follows the recommendations in the ARRIVE guidelines (PLoS Bio 8(6), e1000412,2010). We also followed guidelines issued by the National Institutes of Health (NIH) and the Society for Neuroscience (SfN) to minimize the number of animals used and their suffering. All animal experiments conformed to protocols approved by the University Texas Health Science Center at San Antonio (UTHSCSA) Institutional Animal Care and Use Committee (IACUC). Protocol numbers are 20190114AR, 20220064AR and 20220069AR.

### Key reagents and mouse lines

Total number of 106 mice were used in this study. Mice were housed under controlled conditions (≈22 °C), relative humidity 40–60%, and a 12-h light–dark cycle with lights on at 7:00 a.m. Food and water were available ad libitum in their home cages. Experiments were conducted on wild-type adult (2–4-months-old) C57Bl/6 male mice, which were purchased from Jackson Laboratory (Bar Harbor, ME). The Ccr2^RFP^/Cx3cr1^GFP^ (Stock No: 032127) mouse line on the B6.129 background was purchased from the Jackson Laboratory (Bar Harbor, ME). Crude collagenase (Col) type 2 (> 125 units per mg; Cat: LS004214) preparations were acquired from Worthington (Lakewood, NJ) and complete Freund’s adjuvant (CFA; Cat: F5881) was obtained from Millipore-Sigma (St. Louis, MO).

### Masseter muscle (MM) injection

Left side MM was injected with 10 μλ collagenase type 2 (Col) or CFA (1:1 = CFA: PBS). These injections were performed under isoflurane anesthesia. The area of injection was swabbed with 70% alcohol beforehand. The site for injection was identified by palpating the zygomatic arch and the body of the mandible. A 30-gauge needle was inserted into the point inferior to the posterior third of the zygomatic arch and midway between the zygomatic arch and the body of the mandible. The needle was advanced in an anterior direction in about 3 mm. After a gentle aspiration, solutions were injected. PBS served as vehicle control.

### Measurement of orofacial hypersensitivity

The mechanical hypersensitivity of the left side of orofacial region was assessed as previously described ^28^. To avoid any anticipatory movement from mice during von Frey application. The mice were habituated to small cages for 3 days, then habituated to an area on a table and experimenters hand for an additional 3 days. During the last 3 days, mice were also habituated to von Frey filament (0.07–0.6 g) stimulations. Measurements with von Frey were conducted on the animals kept in hands or freely moving on table. Habituated naive mice that did not respond to 0.6 g von Frey filaments, which is considered baseline, were selected for the experimental procedure ^67^. Experimental mice were probed with 0.008–0.6 g filaments to the skin above MM using an up-down approach ^28^. Intervals between von Frey filament applications were varied but > 30 s. Mechanical thresholds were assessed and calculated as previously described ^28,67^.

### RNA seq transcriptomic data generation, analyses, and statistics

MM was dissected from left side. Dissected MM included deep, middle and superficial parts, and maximally excluded tendons attaching MM to zygomatic and mandible. Fresh MM tissues were homogenized in Rn-easy solution (Qiagen) using a Bead Mill Homogenizer (Omni International, Kennesaw, GA). Extraction of RNA, RNA quality and integrity control, cDNA library preparation with oligo-dT primers following the Illumina TruSeq stranded mRNA sample preparation guide (Illumina, San Diego, CA), and sequencing procedure with Illumina HiSeq 3000 platform with 30–50 × 10^6^ bp reading depth were previously described in detail ^28,29^. Total RNA quality of between 7.5 and 9 were used in RNA-seq experiments. This is a critical point, since power analysis is based on these numbers for quality of total RNA.

Post-sequencing de-multiplexing with CASAVA, the combined raw reads were aligned to mouse genome build mm9/UCSC hg19 using TopHat2 default settings and differentially expressed genes (DEGs) were identified using DESeq2 after performing median normalization, also previously described in detail ^28,29^.

Quality control statistical analysis of outliers, intergroup variability, distribution levels, PCA, and hierarchical clustering analysis were performed to statistically validate the experimental data. Multiple correction test was performed with the Benjamini–Hochberg procedure and adjusted *P*-value (Padj) was generated. If not specified in the text, criteria for selecting DEGs were expression levels with RPKM > 1, fold-change (FC) > 2, and statistically significant DEGs with Padj < 0.05. Venn diagrams were generated using https://bioinfogp.cnb.csic.es/tools/venny/. Genes were clustered according to biological processes using the PANTHER software (http://www.pantherdb.org/).

### Flow cytometry

Flow cytometry was used to assess immune cell profiles in MM biopsies. To eliminate the contributions of immune cells from blood, mice were perfused with cold PBS prior to tissue dissections. Single-cell suspensions from MM biopsies were generated by treating tissues for 70 min at 37 °C with 250 g/ml Liberase (Millipore-Sigma) and 100 g/ml dispase II (Millipore-Sigma), washing with DMEM media containing 5% FCS, triturating with Pasteur pipettes, and then filtering through a 70 μm strainer. Single-cell suspensions were resuspended and centrifuged in isotonic 40% Percoll (Cytiva, Sweden) to enrich live immune cells. Cell suspensions were first stained for viability using Zombie NIR™ Fixable Viability Kit (BioLegend, San Diego, CA) for 20 min at room temperature in PBS combined with FcR blocking antibody (1 μg, clone 2.4G2, BioXCell, Missouri, TX) to block non-specific binding. Cells then were washed with 2% FBS/PBS and stained with antibodies against surface antigens for 30 min on ice. Fluorochrome-conjugated antibodies against mouse CD45 (clone 30-F11), CD3 (145-2C11), B220 (RA3-6B2), CD11b (M1/70), CD64 (X54-5/7.1), CD11c (N418), MHC-II (M5/114.15.2), Ly-6G (1A8), Ly-6C (KH1.4) were purchased from BioLegend (San Diego, CA), eBioscience (San Diego, CA) or BD Biosciences (San Jose, CA). Flow cytometry was performed using Celesta or LSRII cytometer (BD Biosciences; San Jose, CA). Data was analyzed using FlowJo LLC v10.6.1 software. The gating strategy used to select immune populations in MM was done as previously described ^68^. Live CD45^+^ cells were gated using the markers listed below to define cell populations: neutrophils (Nph, CD11b^+^/Ly6G^+^); macrophages (Mph, CD11b^+^/MHCII^hi^/CD64^+^); inflammatory Mph (iMph, CD11b^+^/MHCII^hi^/CD64^+^/Ly6C^+^); monocytes (Mo, CD11b^+^/MHCII^lo^/SSC^lo^/CD64^+^); inflammatory Mo (iMo, CD11b^+^/MHCII^lo^/SSC^lo^/CD64^+^/ Ly6C^hi^); B cells (B, B220^+^/CD11b^-^/CD11c^-^); T cells (T, CD3^+^/CD11b^-^/CD11c^-^), NK (Natural killer cells NK1.1^+^TCRb^-^), CD11b^+^ DCs (Dendritic cells; CD11b^+^CD64^-^CD24^hi^MHCII^hi^CD11c^+^); and CD11b^-^ DCs (CD11b^-^CD64^-^CD24^hi^MHCII^hi^CD11c^+^).

### Immunohistochemistry (IHC)

MM were dissected from 4% paraformaldehyde perfused Ccr2^RFP^/Cx3cr1^GFP^ mice, fixed additionally with 4% paraformaldehyde for 20 min, cryoprotected with 10% and then 30% sucrose in phosphate buffer and then embedded in Neg-50™ (Richard Allan Scientific, Kalamazoo, MI). Cryo-sections of MM (30–35 μm) were collected for IHC. IHC was carried out as previously described^28,69^. The following antibodies were used in the study: anti-rat monoclonal S100A8 (Cat: MAB3059; R&D Systems; Minneapolis, MN) and anti-goat Iba1 (Cat: NB100-1028; Novus; Centennial, CO). For non-conjugated primary antibodies, sections were incubated with species-appropriate donkey Alexa Fluor secondary antibodies (1:200; Jackson Immuno-Research, West Grove, PA). Control IHC was performed on tissue sections processed as described but either lacking primary antibodies or lacking primary and secondary antibodies.

Z-stack IHC images were acquired from 3 to 5 independent tissue sections from 3 animals using a Keyence BZ-X810 All-in-One Fluorescent Microscope (Keyence, Itasca, IL) or a Nikon Eclipse 90i microscope (Melville, NY, USA) equipped with a C1si laser scanning confocal imaging system. Images were processed with NIS-elements software (Nikon Instruments, Melville, NY) or Adobe Photoshop CS2 software. The scale of microphotographs were placed by NIS-elements or Keyence software.

### Statistical analyses

Sample size calculations were based on our previous experience in RNA-seq and behavioral experiments on linear and inbred C57-black mice^29,70^. Accordingly, in behavioral experiments, we estimate that 6–8 mice will achieve > 80% statistical power to achieve *P* < 0.05 difference in measurement of hypersensitivity. For bulk RNA-seq, a sample size of 3–4 mice per group, using false discovery rate (FDR) of 0.05, will achieve > 80% statistical power to detect a two-fold change in gene expression with an estimated standard deviation of 0.5. We also assume 1% of genes are differentially expressed in the system, or 100 differentially expressed genes among the typical 10,000 expressed genes in tissues profiled. Using Scotty^71^, we estimate that 40 million reads/samples are needed.

GraphPad Prism 8.0 (GraphPad, La Jolla, CA) was used for statistical analysis. Data are presented as mean ± standard error of mean (SEM). Differences between groups were assessed by chi-square analysis with Fisher’s exact test, unpaired t-test, or 1-way ANOVA with Bonferroni’s post-hoc test. A difference is accepted as statistically significant when *P* < 0.05. Interaction F ratios, and the associated p values are reported.

### Supplementary Information

Supplementary Figures.

## Data Availability

RNA-seq data has been deposited to GEO Accession, the number is GSE190183. Supplementary excel files show the raw gene readings/counts per gene for all our sequencing experiments and RPKM data for each sample. These supplementary files are “*Veh vs 1d post-CFA (MM; G-L columns are RPKM)*” for RNA-seq from male mouse MM at 1d post-CFA intramuscular; “*Veh vs 5d post-CFA (MM; G-L columns are RPKM)*” for RNA-seq from male mouse MM at 5d post-CFA intramuscular; “*Veh vs 1d post-Col 0.2U (MM; G-O columns are RPKM)*” for RNA-seq from male mouse MM at 1d post-Col (0.2U) intramuscular; “*Veh vs 5d post-Col 0.2U (MM; G-O columns are RPKM)*” for RNA-seq from male mouse MM at 5d post-Col (0.2U) intramuscular; “*Veh vs 1d post-Col 10U (MM; G-O columns are RPKM)*” for RNA-seq from male mouse MM at 1d post-Col (10U) intramuscular; “*Veh vs 6d post-Col 10U (MM; G-N columns are RPKM)*” for RNA-seq from male mouse MM at 6d post-Col (10U) intramuscular; “*Veh vs 14d post-Col 10U (MM; G-O columns are RPKM)*” for RNA-seq from male mouse MM at 14d post-Col (10U) intramuscular injection.
