# Supplementary material for: Associations of tissue damage induced inflammatory plasticity in masseter muscle with the resolution of chronic myalgia
Source: Sci Rep. 2023 Dec 12;13:22057. doi: 10.1038/s41598-023-49280-1 (PMC10716154; doi:10.1038/s41598-023-49280-1)
Supplement: Supplementary file 1 — Supplementary Figures. [file 41598_2023_49280_MOESM1_ESM.pdf]

Legends to Supplementary Figures:

Supplementary Figure 1: Raw data on gating strategy for flow cytometry

Gating strategy for single-cell suspension from the MM at 1d post-vehicle (Veh) or CFA single intramuscular treatment. The strategy shows live singlets and different immune cells were gated from single-cell suspension. Abbreviations are SSC-A - side angle scattered area; FSC-A - forward angle scattered area; $CD45^+$ - immune cells; Mo - monocytes, Nph – neutrophils, CD3 – T-cells.

Supplementary Figure 2: Immune cell profiles in CFA and Col treated MM at post-hypersensitivity resolution time points.

(A) Immune cell counts per 10^4 $CD45^+$ cells in male MM at 5d post-vehicle (Veh) or CFA single intramuscular treatment. (**B**) Immune cell counts per $CD45^+$ cells in MM at 14d post-vehicle (Veh), Col (0.2U) or Col (10U) single intramuscular treatment. DCs – dendritic cells; Mph – macrophages; iMph - inflammatory macrophages; Mo – monocytes; iMo - inflammatory monocytes; NK – natural killer cells; B – B-cells; T – T-cells and Neu – neutrophils. Statistic is 2-way ANOVA (* $p<0.05$; # $p<0.0001$; n=3-6).

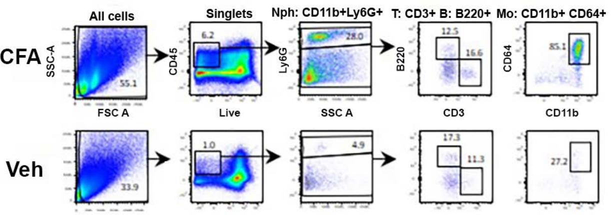

Supplementary Figure 1

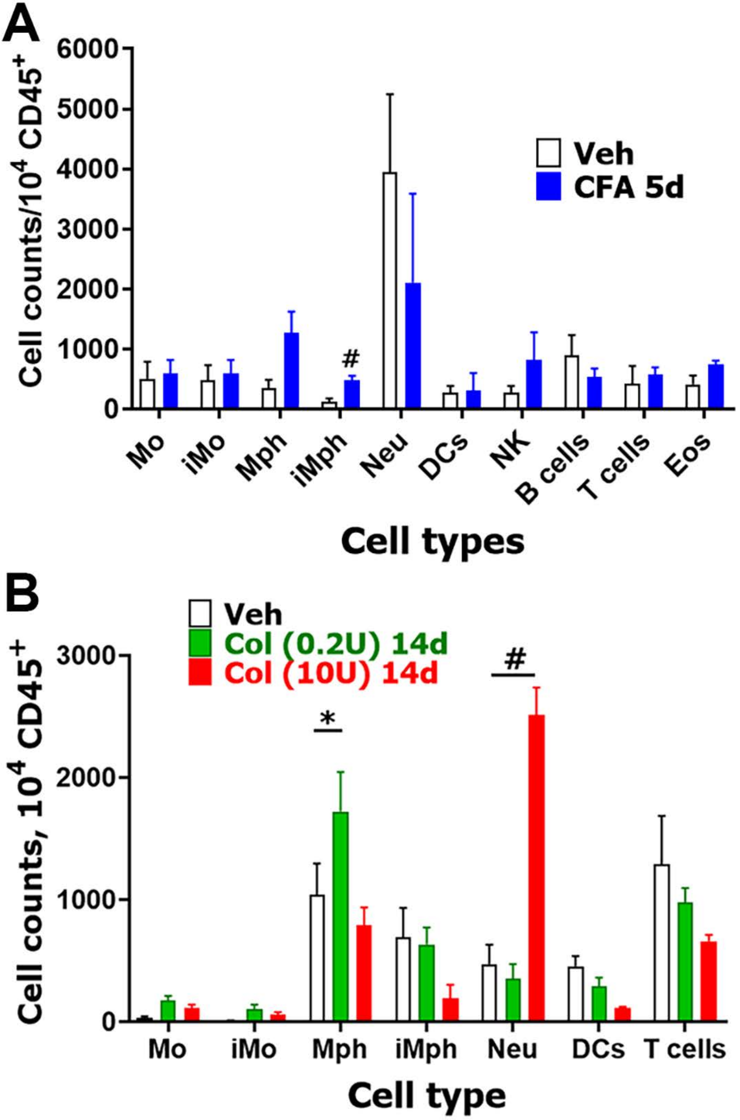

Supplementary Figure 2
